# Modulation of inflammatory responses by fractalkine signaling in microglia

**DOI:** 10.1371/journal.pone.0252118

**Published:** 2021-05-21

**Authors:** Koichi Inoue, Hiroyuki Morimoto, Masahiro Ohgidani, Takatoshi Ueki

**Affiliations:** Department of Integrative Anatomy, Nagoya City University Graduate School of Medical Sciences, Nagoya, Japan; Lewis Katz School of Medicine, Temple University, UNITED STATES

## Abstract

Reactive microglia are suggested to be involved in neurological disorders, and the mechanisms underlying microglial activity may provide insights into therapeutic strategies for neurological diseases. Microglia produce immunological responses to various stimuli, which include fractalkine (FKN or CX3CL1). CX3CR1, a FKN receptor, is present in microglial cells, and when FKN is applied before lipopolysaccharide (LPS) administration, LPS-induced inflammatory responses are inhibited, suggesting that the activation of the FKN signal is beneficial. Considering the practical administration for treatment, we investigated the influence of FKN on immunoreactive microglia using murine primary microglia and BV-2, a microglial cell line. The administration of LPS leads to nitric oxide (NO) production. NO was reduced when FKN was administered 4 h after LPS administration without a change in inducible nitric oxide synthase expression. In contrast, morphological changes, migratory activity, and proliferation were not altered by delayed FKN treatment. LPS decreases the CX3CR1 mRNA concentration, and the overexpression of CX3CR1 restores the FKN-mediated decrease in NO. CX3CR1 overexpression decreased the NO production that is mediated by LPS even without the application of FKN. ATP and ethanol also reduced CX3CR1 mRNA concentrations. In conclusion, the delayed FKN administration modified the LPS-induced microglial activation. The FKN signals were attenuated by a reduction in CX3CR1 by some inflammatory stimuli, and this modulated the inflammatory response of microglial cells, at least partially.

## Introduction

Microglia are immune cells present in the central nervous system. Early evidence has indicated that microglia are associated with neuropsychiatric disorders, including schizophrenia and chronic neurodegenerative disorders such as amyotrophic lateral sclerosis. Recently, findings have further suggested the involvement of microglia in other pathophysiological events such as the formation of neuropathic pain and synaptic remodeling [1–4]. Reactive microglia are observed with *in vivo* PET tracers in psychiatric patients [5], and the regulation of microglial activity is expected to become a therapeutic target soon. Microglia become pro-inflammatory following various forms of stimulation, which results in the upregulation of inflammatory mediators such as inducible nitric oxide synthase (iNOS) and TNFα [6, 7]. iNOS ultimately generates reactive oxygen species that cause neuronal injury. Cytokines, including TNFα, stimulate other immune cells, which are involved in the development of neurological disorders. Microglial activity is altered by modulatory factors such as fractalkine (FKN or CX3CL1) [8].

FKN is a chemokine associated with cell migration and immune responses, first generated as a membrane protein. Membrane-bound FKN is digested enzymatically by selected members of metalloproteinases, and a soluble extracellular region is released out of the cell surface [3, 9, 10]. Its receptor, CX3CR1, is expressed mainly in immune cells, including microglial cells, and the receptor-mediated immune responses of FKN significantly modulate microglial function. Several studies indicate that CX3CR1 plays an anti-inflammatory role through microglial activity [11, 12], whereas some report that CX3CR1 deficiency promotes stress resilience in the brain [13–15].

Thus, FKN may be leveraged in therapeutic strategies for neurological diseases that are caused by reactive microglia. In terms of the practical mechanisms of this kind of therapy, FKN should affect the regulation of reactive microglia. A recently developed CX3CR1 inhibitor revealed the possibility that delayed administration of FKN plays a role in the suppression of pro-inflammatory microglia [16, 17]. This suggests that activation of FKN signaling inhibits even reactive microglia, but the details have not been studied at the cellular level *in vitro*. We investigated the effect of FKN on microglia that were stimulated in advance and the mechanism underlying the effect of FKN signaling on microglial activation.

## Materials and methods

### Reagents and antibodies

The following reagents and antibodies were used: Isogen (Nippon Gene); lipopolysaccharide (LPS, Escherichia coli O111:B4, Sigma-Aldrich); soluble murine FKN (Cat. #228-20032-2, RayBiotech); protease inhibitor cocktail (Cat. # S8820, Sigma-Aldrich); 2,3-diaminonaphthalene (Cat. # 341–07021, Dojindo); mouse monoclonal antibodies against iNOS (Cat. # 610328, BD Biosciences), GAPDH (Cat. # 600004-1-Ig, Proteintech Group Inc.), CD11b (Cat. # 101202, BioLegend) and hemagglutinin (Cat. # M180-3S, HA, MBL); rabbit polyclonal antibodies against actin (Cat. # A2066, Sigma-Aldrich) and CX3CR1 (Cat. # 14-6093-81, eBioscience), a goat polyclonal antibody against GFAP (Cat. # ab53554, Abcam).

### Cell culture

A microglial cell line, BV-2, was grown on Dulbecco’s modified eagle medium (DMEM) with 5% fetal bovine serum (FBS) and antibiotics. A vascular endothelial cell line, F-2 [18], was cultured in DMEM with 10% FBS and antibiotics.

The primary microglial culture has been described elsewhere [19]. The experiments were approved by Ethics Committee of the Nagoya City University Graduate School of Medical Sciences and conducted following the Guidelines for Use of Laboratory Animals (Approval No. H28M-048). Briefly, P0-1 ICR mice (SLC, Hamamatsu) were anesthetized with isoflurane, followed by cervical dislocation and the isolation of cerebral cortices. After the meninges were removed, the cortices were cut into small pieces and soaked in phosphate-buffered saline (PBS). The tissues were sedimented and washed with DMEM three times and incubated with Trypsin/EDTA and 100 μg/ml DNase I (Roche). The tissue was triturated gently and filtered through a cell strainer. The cells were plated on poly-L-lysine coated-flask and DMEM containing 10% FBS. The microglial cells were isolated for 7–14 days in a gentle shaking cycle and then transferred to appropriate culture plates. Approximately 99% of the cells were found to be CD11b-positive (a marker for microglia) (S1 Fig).

### Plasmid construction and transfection

The RNAs of primary microglia and the BV-2 cells were extracted using Isogen (Nippon Gene). The cDNAs were synthesized from the extracted RNAs using oligo (dT)_15_ and reverse transcriptase (Toyobo) reactions.

Murine CX3CR1 and taurine transporter TauT (as a negative control) were cloned and added to the pCAGGS vector (provided by Dr. Miyazaki, Osaka University) with a hemagglutinin (HA) tag. For transfection, 1 × 10^6^ cells were electroporated with 10 μg of plasmids using an electroporator NEPA21 Type II according to the manufacturer’s instructions (NEPA Gene).

### Migration assay

The cells were seeded to 12-well plates at a density of 3 × 10^5^ cells/well. The next day, the cell monolayer was scratched with a 1000-μl tip. After three washes with PBS, the cells were either untreated or treated with 500 ng/ml LPS for 4 h, followed by incubation in the absence or presence of 200 ng/ml of FKN for another 24 h. Photographs were obtained, and the cells in the scratched area were counted.

### Proliferation assay

The proliferation assay has been described elsewhere [20]. The cells were plated into 24-well plates at 1 × 10^3^ cells/well. The next day, the cells were treated with the indicated reagents. Images of the cells in randomly selected wells, with nine fields per well, were captured, and continued to be obtained in the same fields at 24-h intervals using IN Cell Analyzer 6000 (GE). The images were deconvoluted using Developer software (GE) to recognize the cells morphologically, and the cells were counted.

### Immunocytochemistry

The cells were fixed with 4% paraformaldehyde in PBS, followed by incubation with 5% horse serum and 0.2% Triton X-100 at room temperature. Subsequently, they were incubated with anti-HA (1:1000), anti-CX3CR1 (1:100), anti-CD11b (1:1000), or anti-GFAP (1:1000) antibodies, followed by fluorescence (Alexa488 and Alexa594)-conjugated secondary antibodies (1:1000, Thermo Fisher Scientific). For DNA staining, the cells were incubated with 4’,6- diamidino-2-phenylindole (DAPI) (Thermo Fisher Scientific).

### Quantitative real-time PCR

After RNA extraction and cDNA synthesis, as described above, quantitative real-time PCR was performed to validate the changes in the expression of the selected genes using SYBR^®^ Premix Ex Taq (Cat. # RR820A, TaKaRa) and the Thermal Cycler Dice Real-Time System (Takara), as described in a previous study [19]. The primer sets have been described in S1 Table.

### Immunoblotting

Immunoblotting was performed as described in previous studies [21, 22]. The cells cultured on 6-well plates were lysed in a lysis buffer (50 mM Tris-HCl, pH 7.5, 100 mM NaCl, 1% Triton X-100, and protease inhibitor). Following centrifugation at 15,000 × g at 4˚C for 30 min, the lysates were collected. Subsequently, the aliquots were mixed with Laemmli sample buffer and boiled at 95˚C for 10 min. The samples were resolved by SDS-PAGE, then electrotransferred to polyvinylidene difluoride membranes. For visualization, the blots were probed with antibodies for iNOS (1:2000), actin (1:1000), or GAPDH (1:5000) and detected using horseradish peroxidase-conjugated secondary antibodies (1:2000; Promega) and an ECL kit (Bio-RAD).

### Measurement of nitric oxide (NO) metabolites

The production of NO was assessed by measuring nitrite, a stable product of NO, using fluorometric reagent 2,3-diaminonaphthalene [23, 24]. The cells were either untreated or treated with 500 ng/ml LPS for 4 h, followed by incubation in the absence or presence of 200 ng/ml FKN for another 24 h. Subsequently, 100 μl of the samples were transferred to 96-well plates and incubated with 10 μl of fresh 2,3-diaminonaphthalene solution (50 μg/ml in 0.62 N HCl) for 10 min at room temperature. The reactions were terminated with 5 μl of 2.8 N NaOH. The formation of 2,3-diaminonaphthotriazole was evaluated using a fluorescent multi-well plate reader (SpectraMax Gemini, Molecular Devices) with excitation/emission at 365/450 nm. The fluorescence signal was digitized and analyzed using SoftMax Pro software (Molecular Devices).

### ELISA

A mouse FKN ELISA kit was used (MCX310, R&D Systems). The cells were incubated in 24-well plates, and the ELISA plates were filled with 50 μl media following the manufacturer’s instructions. Absorbance was measured at 450 nm, with 570 nm as a reference, using a microplate reader (Molecular Devices).

### Statistical analysis

Data are presented as means ± SEM. Kaleidagraph (Synergy Software) and SPSS (SPSS Inc.) were used for statistical analysis. The data were assumed to be normally distributed, and the differences between the groups were compared using an unpaired Student’s *t*-test, a one-way ANOVA (Figs 2B, 2C, and 4). Bonferroni’s test was used to compensate for multiple experimental procedures. For Fig 3B, A two-way ANOVA was used to examine the two main effects of the treatment (three levels: control, LPS or LPS + FKN) and the overexpression of CX3CR1-HA (two levels: absence or presence) as between-subject factors on NO production. Their interaction effects were also analyzed. Effect sizes were estimated by calculating the value of η^2^ for data analyzed by two-way ANOVA. Follow-up pairwise comparisons were performed using the Sidak’s method. A p value < 0.05 was considered statistically significant.

## Results

### The effect of FKN on NO production induced by LPS

**Fig 1:**
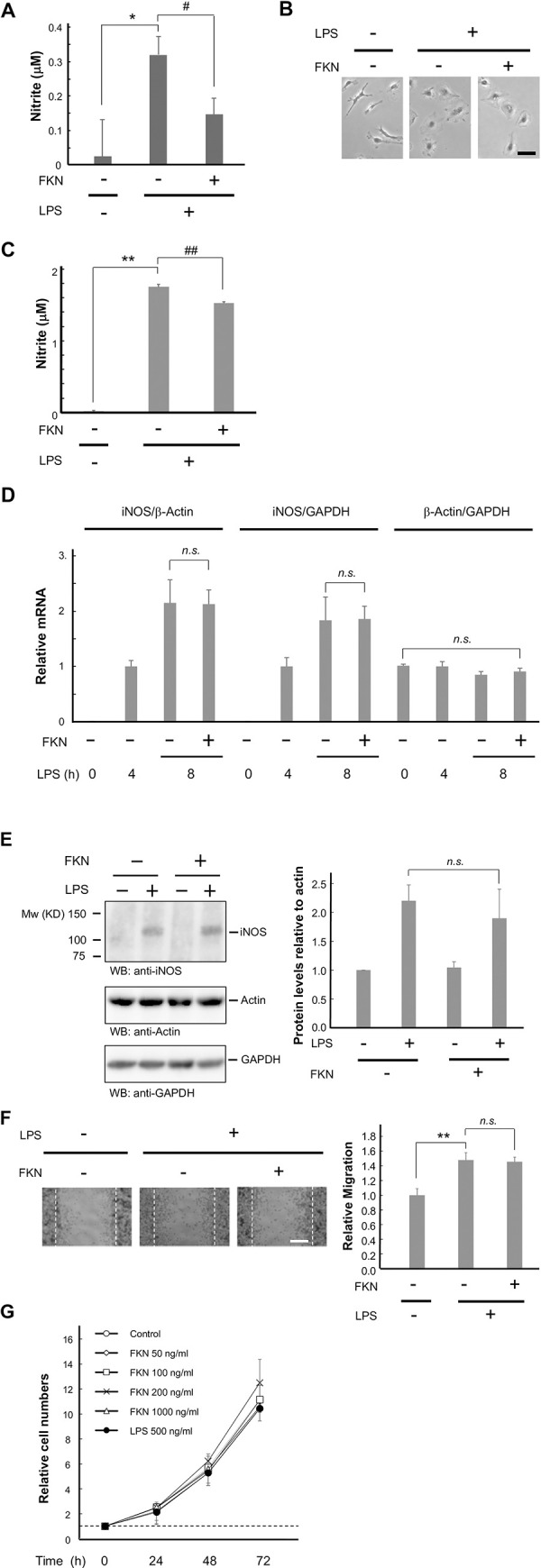
FKN attenuates microglial NO products when pre-stimulated with LPS. Primary microglia (A and B) and BV-2 cells (C to G) were treated with 500 ng/ml LPS. Four hours later, 200 ng/ml FKN was applied, followed by another 4 h (D) or 20 h (except D) of incubation. (A) The media were obtained, and NO production was measured by assessing nitrite levels. n = 4–7. * p < 0.05 vs. control, # p < 0.05 vs. LPS-treated cells, Student’s *t*-test. (B) Images show the morphology 24 h following LPS administration. Scale bar = 20 μm. (C) The media were obtained and NO was measured, as described in (A). n = 3–4. ** p < 0.01 vs. control, ## p < 0.01 vs. LPS-treated cells, Student’s *t*-test. (D) Quantitative real-time PCR was performed to monitor the mRNA concentrations of iNOS in comparison with those of GAPDH or β-actin mRNA in cells treated with the indicated reagents. n = 6. (E) BV-2 cell lysates were analyzed by immunoblotting with the indicated antibodies. The bar chart indicates the relative iNOS protein concentration as compared with the densities determined in the LPS-positive and FKN-negative cells. n = 4. (F) BV-2 cells were grown as a confluent monolayer and scratched, followed by incubation with or without LPS. FKN was administered 4 h later, and migration was evaluated 20 h later. The bar chart indicates the relative cell numbers in comparison with those determined for the control cells. n = 4. ** p < 0.01 vs. control. Scale bar = 50 μm. (G) The cells were seeded and photos of the same fields were obtained every 24 h. The summary graph shows the fold changes in the normalized cell numbers of the indicated cells. n = 4 independent dishes.

Although it has been reported that FKN attenuates pro-inflammatory microglial activity [25–27], it is unclear whether FKN suppresses the activity of reactive microglia. To address this issue, LPS was first added to induce microglial activation, followed by the addition of FKN 4 h later to murine primary microglial cells. NO production was induced by LPS, and the delayed administration of exogenous FKN decreased it (control, 0.03 ± 0.11 μM; LPS, 0.32 ± 0.05 μM; LPS + FKN, 0.14 ± 0.05 μM) (Fig 1A). Consistent with previous reports [28, 29], LPS administration induced morphological changes from the resting forms with short, branched processes to relatively flattened reactive forms. We assessed the effect of FKN on their shapes, but it did not appear to affect them (Fig 1B). We also examined the effect of FKN on NO production in a microglial cell line, BV-2, and a similar tendency was observed (control, 0.01 ± 0.01 μM; LPS, 1.75 ± 0.03 μM; LPS + FKN, 1.52 ± 0.02 μM) (Fig 1C). We attempted to perform the experiments mainly on BV-2 cells.

To determine whether the FKN-mediated reduction in NO is attributable to iNOS concentration, the gene expression of iNOS was evaluated. Four hours after LPS treatment, FKN was added. The subsequent 4-h treatment with FKN did not change the iNOS mRNA concentrations against GAPDH mRNA (FKN -, 1.83 ± 0.42; FKN +, 1.86 ± 0.23) (Fig 1D). As LPS treatment can affect the gene expression of housekeeping genes [30], we also compared the expressions of iNOS and β-actin, another common housekeeping gene. The β-actin concentrations did not significantly change compared to GAPDH (FKN -, 0.85 ± 0.06; FKN +, 0.91 ± 0.06), and the effect of FKN on iNOS gene expression was not detected (FKN -, 2.15 ± 0.42; FKN +, 2.13 ± 0.26) (Fig 1D). Similar to the mRNA concentrations, the iNOS protein concentrations did not change (FKN -, 2.20 ± 0.27; FKN +, 1.90 ± 0.51) (Fig 1E and S2 Fig). These results suggest that FKN decreases NO production without affecting iNOS protein concentrations.

### The effect of FKN on migration and proliferation

We determined the effect of the delayed administration of FKN on LPS-induced microglial migration. The BV-2 cells were scratched with a pipette tip. It was microscopically observed that LPS accelerated migration, but this was not altered by FKN (FKN -, 147.8 ± 10.1%; FKN +, 145.6 ± 6.3%) (Fig 1F).

Similarly, LPS and FKN did not affect proliferation in BV-2 cells (200 ng/ml FKN, 1250 ± 190% on day 4; 500 ng/ml LPS, 1040 ± 130% on day 4) (Fig 1G).

### The effect of LPS on the gene expression of the FKN receptor

**Fig 2:**
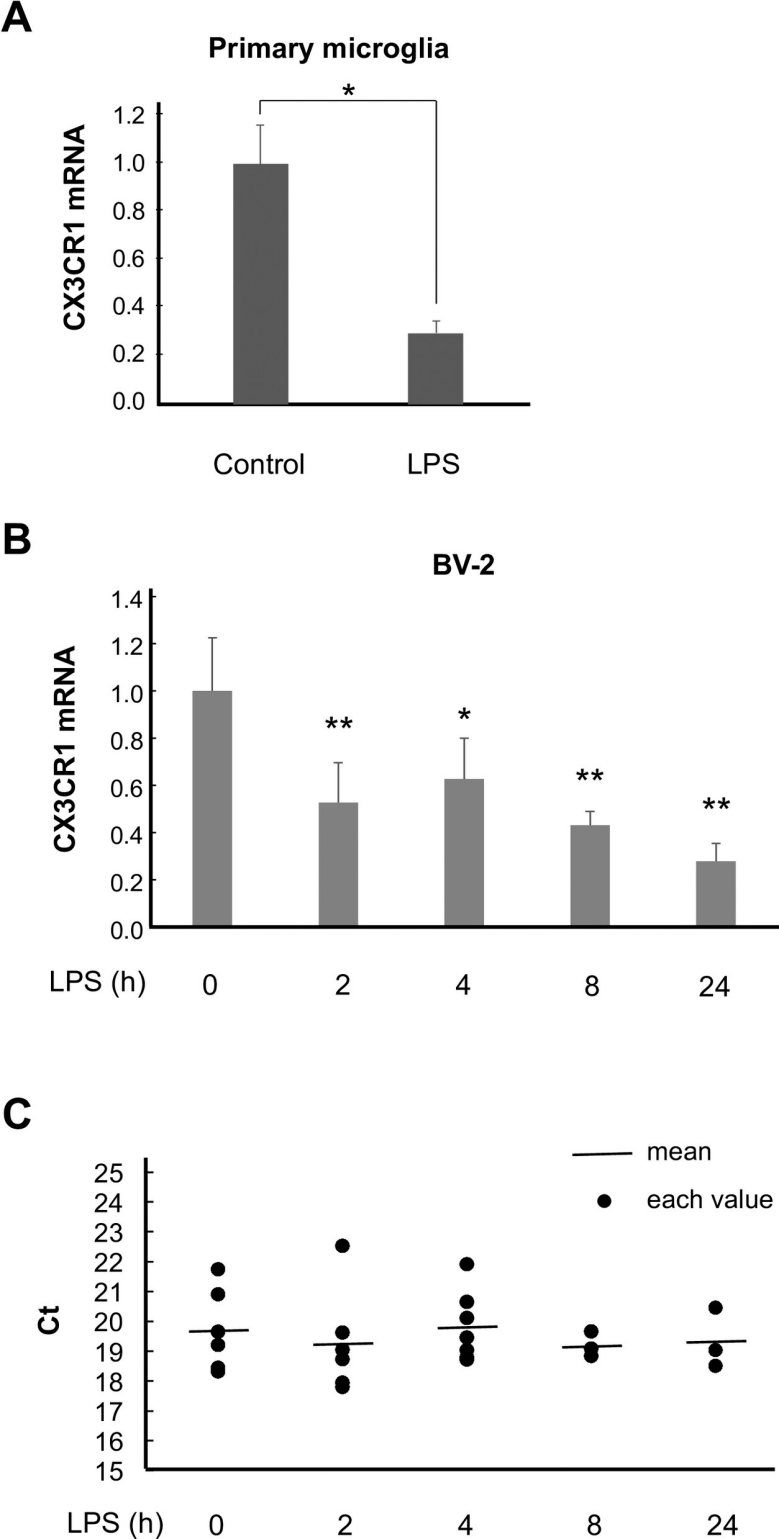
LPS downregulates CX3CR1 gene expression. (A) Primary microglia were treated with LPS for 4 h, and the cells were subjected to quantitative real-time PCR. n = 3. * p < 0.05 vs. control, Student’s *t*-test. (B) BV-2 cells were treated with LPS for the indicated times and subjected to quantitative real-time PCR. n = 3–7. * p < 0.05, ** p < 0.01 vs. 0 h, one-way ANOVA followed by Bonferroni’s test. (C) Ct values of GAPDH in (B) were indicated with black circles. The mean values were indicated with black bars. n = 3–7. p = 0.91 vs. 0 h, one-way ANOVA.

FKN signaling in the nervous system has been investigated, and the gene expression of the FKN receptor, CX3CR1, has been reported to be downregulated by LPS *in vivo* and in BV-2 cells [31]. A similar observation was made in primary microglia (control, 100.0 ± 16.0%; LPS, 29.5 ± 5.1%) (Fig 2A). We evaluated the time course of the effect of LPS on BV-2 cells. The mRNA concentration of CX3CR1 compared with that of the control was reduced as early as 2 h following LPS treatment, and the reduction persisted for up to 24 h (0 h, 100.0 ± 22.5%; 2 h, 52.6 ± 17.0%; 4 h, 62.6 ± 17.4%; 8 h, 43.1 ± 5.8%; 24 h, 27.8 ± 7.6%) (Fig 2B). Meanwhile, GAPDH concentrations were not affected (Ct values of GAPDH at 0 h, 19.71 ± 0.56; 2 h, 19.23 ± 0.71; 4 h, 19.81 ± 0.44%; 8 h, 19.19 ± 0.26%; 24 h, 19.34 ± 0.58%) (Fig 2C).

### Improvement of the FKN signal by the overexpression of CX3CR1

**Fig 3:**
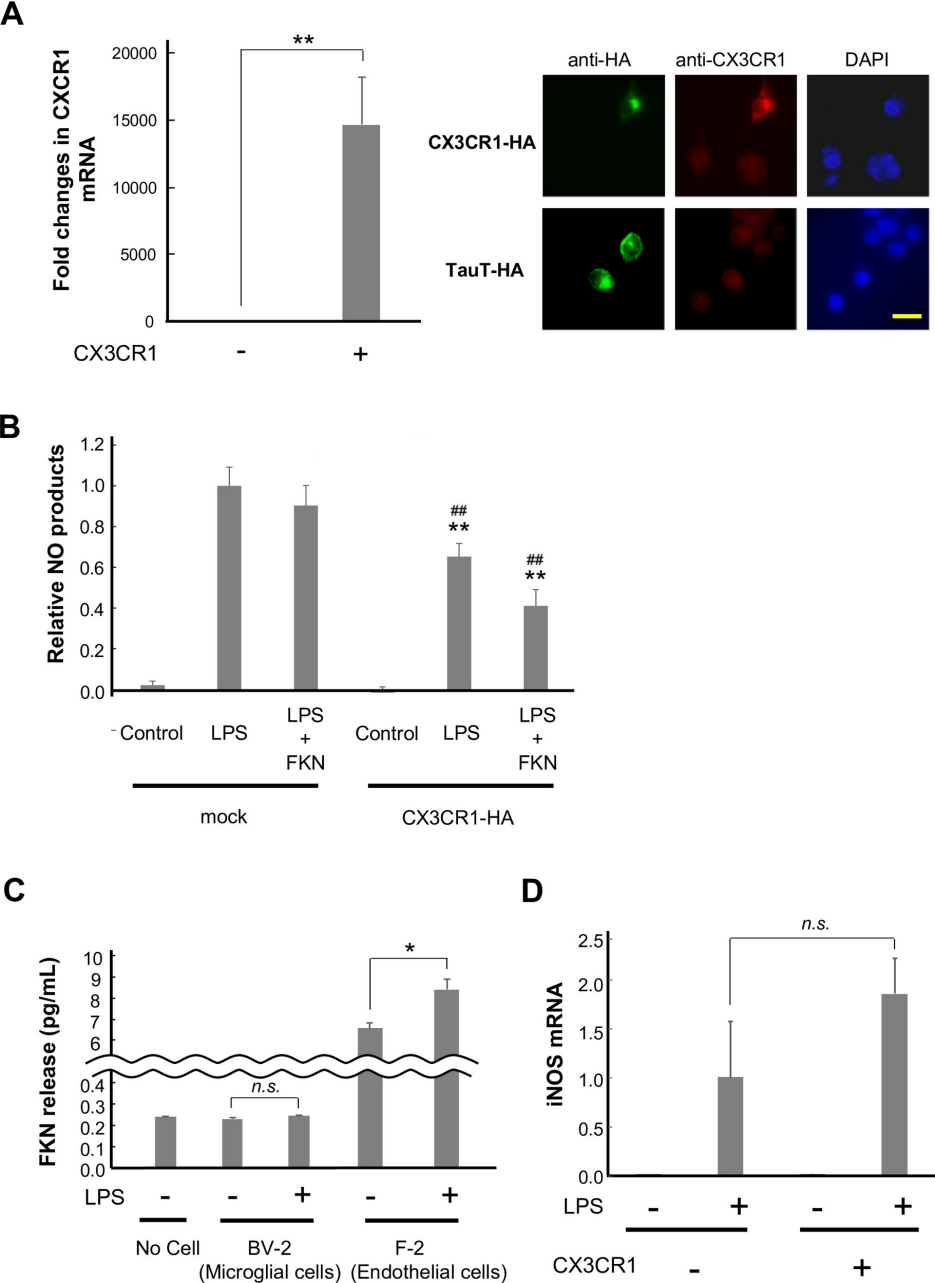
CX3CR1 overexpression attenuates LPS-induced inflammatory responses. (A) CX3CR1-HA-expressing or mock plasmids were transfected into the BV-2 cells. The cells were collected 2 days later, and the RNAs were extracted, followed by cDNA synthesis and quantitative real-time PCR. n = 3. The images show the detection of exogenous CX3CR1 using antibodies for CX3CR1 and HA. An HA-tagged taurine transporter was used as a negative control. The cells were stained with DAPI. Scale bar = 20 μm. (B) CX3CR1-HA-expressing or mock plasmids were transfected into the BV-2 cells. After 2 days, the cells were treated with LPS, and FKN was administered 4 h later. Twenty hours later, the media were collected, and the NO concentration was measured. n = 3–4. ** p < 0.01 vs. mock, ## p < 0.01 vs. control, two-way ANOVA followed by Sidak’s multiple comparison test. (C) The cells were either untreated or treated with LPS for 24 h. The media were collected, and FKN was measured using ELISA. n = 3–4. * p < 0.05 vs. LPS -, Student’s *t*-test. (D) A CX3CR1-HA-expressing plasmid or a mock vector was transfected into the BV-2 cells. Two days later, they were treated with LPS for 4 h, and the RNAs were extracted, followed by cDNA synthesis and quantitative real-time PCR. n = 3.

To examine whether the downregulation of CX3CR1 impairs the anti-inflammatory response to FKN, the BV-2 cells were transfected with CX3CR1. Two days after the transfection, LPS was added, followed by FKN application 4 h later. Following the transfection, CX3CR1 was overexpressed, and its exogenous form was detected immunohistochemically (relative CX3CR1 mRNA, control, 1.0 ± 0.4; LPS, 14600 ± 3600%) (Fig 3A). A two-way ANOVA revealed that the main effects of CX3CR1 overexpression [*F*_(1, 19)_ = 22.65, p < 0.01, η^2^ = 0.618] and treatment [*F*_(2, 19)_ = 66.30, p < 0.01, η^2^ = 0.904] were significant, such that CX3CR1 decreased the effect of treatment (p < 0.01) and LPS induced NO production (p < 0.01) (relative NO production, CX3CR1-HA(-)/control, 0.02 ± 0.02; CX3CR1-HA(-)/LPS, 1.00 ± 0.09; CX3CR1-HA(-)/LPS + FKN, 0.90 ± 0.10; CX3CR1-HA(+)/Control, -0.01 ± 0.03; CX3CR1-HA(+)/LPS, 0.65 ± 0.07; CX3CR1-HA(+)/LPS + FKN, 0.41 ± 0.08, Fig 3B). The treatment × CX3CR1 overexpression interaction had significance [*F*_(2, 19)_ = 4.45, p = 0.032, η^2^ = 0.389], such that FKN exhibited a decreasing trend in LPS-induced NO production in CX3CR1-transfected cells (p = 0.104), but not in mock vector-transfected cells (p = 0.76). Thus, the FKN-mediated reduction in NO appeared to be greater compared with the mock-transfected cells.

If FKN is released constantly from microglial cells, it may be reasonable that exogenous CX3CR1 receives endogenous FKN, which results in the inhibition of NO production, regardless of FKN administration. To address this possibility, the extracellular FKN concentration was investigated using ELISA. As shown in Fig 3C, FKN was not detected even after LPS administration in BV-2 cells (no cells, 0.23 ± 0.01 pg/ml; BV-2/LPS(-), 0.23 ± 0.01; BV-2/LPS(+) 0.24 ± 0.01 pg/ml). To confirm whether our measurement system works, we examined the FKN levels in vascular endothelial cells considering that they are known to produce FKN constantly and that the release is induced by LPS [32]. As expected, they released FKN, and LPS facilitated its release (F-2/LPS(-), 6.60 ± 0.27; F-2/LPS(+) 8.44 ± 0.52 pg/ml) (Fig 3C).

The overexpression of CX3CR1 did not alter iNOS gene expression (relative iNOS expression, CX3CR1-HA(-), 1.00 ± 0.57; CX3CR1-HA(+), 1.84 ± 0.37) (Fig 3D); this rules out the possibility that the decrease in LPS-induced NO production resulted from the reduction in CX3CR1-mediated iNOS.

### The effect of inflammatory stimuli on CX3CR1 gene expression

**Fig 4:**
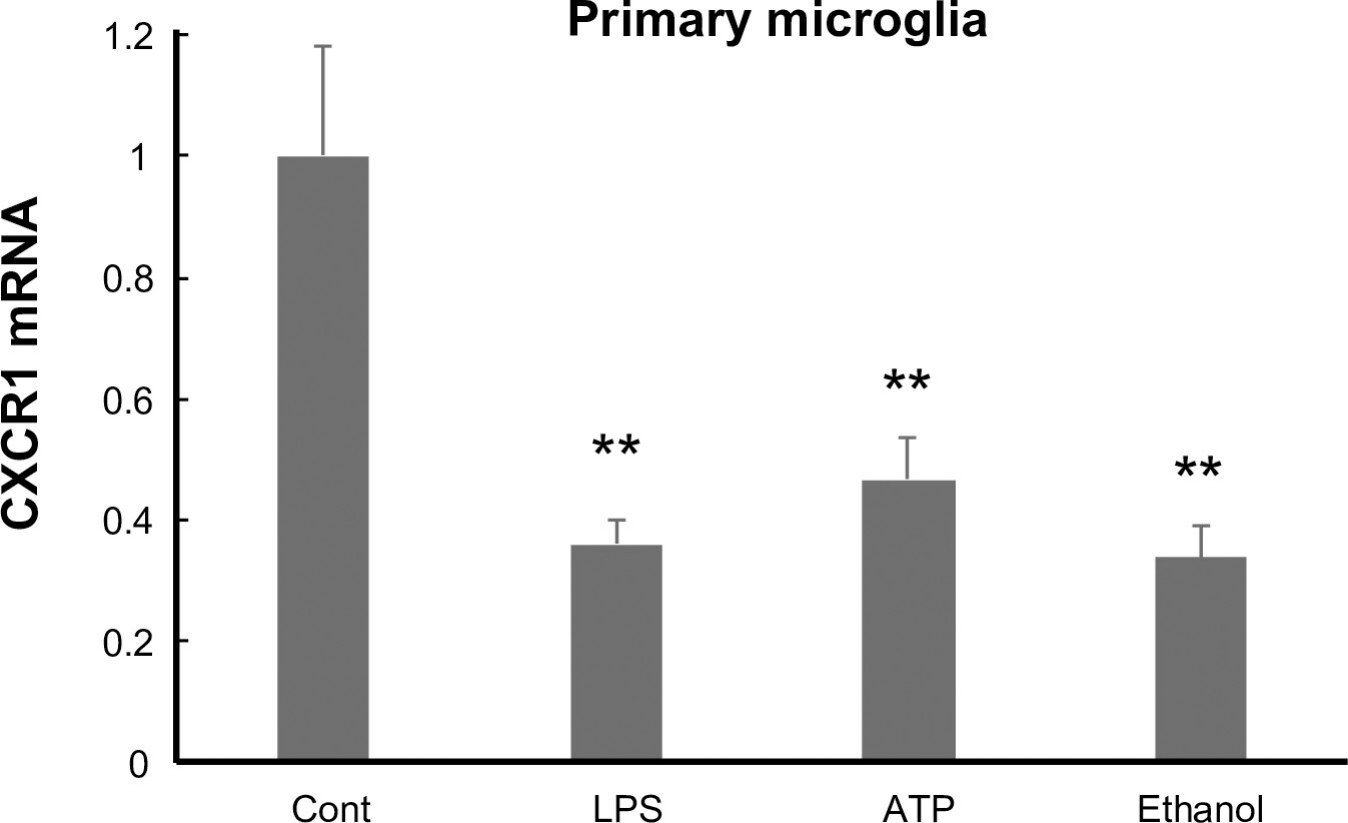
CX3CR1 gene expression is downregulated by inflammatory stimuli. Primary microglial cells were treated with the indicated reagents for 4 h and subjected to quantitative real-time PCR. n = 3–6. ** p < 0.01 vs. control, one-way ANOVA followed by Bonferroni’s test.

To investigate whether FKN signaling is also responsible for other stimulations, the primary microglial cells were treated with some irritants. ATP and ethanol, as well as LPS, lowered CX3CR1 gene expression significantly (control, 100.0 ± 17.9%; LPS, 36.0 ± 3.9%; ATP, 46.7 ± 6.8%; EtOH, 33.7 ± 5.1%) (Fig 4). Taken together, the inflammatory stimuli may attenuate FKN signaling via the downregulation of CX3CR1.

## Discussion

Since the discovery of FKN, intensive efforts have resulted in the recognition of the significant roles of FKN in cellular survival or activity. Its benefits are still being explored in immune cells and other cell types, including endothelial and neuronal cells. Similarly, the roles of FKN have also been reported in microglia, but they were not determined clearly in already activated microglia. Because the attenuation of microglial activity may provide insights into the FKN-related therapeutic strategy, we investigated these aspects.

First, we showed that the NO production triggered by LPS was ameliorated by administering FKN after LPS in microglia. Although the impact of these reagents on NO production varies between primary microglia and BV-2 cells, some studies also show similar trends. For example, primary microglia and BV-2 cells produce NO by LPS/IFN-γ treatment, but the amount of NO in BV-2 cells are about 10-fold greater than that of primary microglia [33]. Thus, this discrepancy could often happen, possibly because of the differences of LPS receptor (TLR4), CX3CR1, intracellular signaling molecules, or currently unknown mechanisms. We speculated that the reduction in NO was due to the decrease in iNOS concentration; therefore, we evaluated the mRNA and protein concentrations in BV-2 cells. The iNOS concentrations were not altered, which was unexpected. Thus, FKN attenuates iNOS activity without changing its protein concentration. The exact mechanism underlying the suppression of iNOS activity is unknown, but the reduction in iNOS activity without a change in the protein concentration has been reported. For example, the lack of arginine, a substrate of iNOS, impairs NO production [34] and the phosphorylation of Tyr151 and Ser745 has been indicated to influence the production of NO by iNOS [35, 36]. CX3CR1 is a G protein-coupled receptor; stimulation of the FKN/CX3CR1 axis results in the activation of signaling pathways, such as the PI3K and MAPK pathways [37, 38]. In addition, recent reports demonstrated that FKN/CX3CR1 activates Src signaling in some cells [39, 40]. Src signaling is responsive to the phosphorylation of Ser745 in iNOS, which inhibits NO producing activity [35]. Although Src is a protein-tyrosine kinase and it does not appear to phosphorylate it directly, Src signaling might be one of plausible candidates for inhibition of iNOS activity. Thus, our observed reduction in NO production may have been independent of the level of expression. This can also happen if FKN directly interacts with iNOS and inhibits its activity; however, this cannot be the case because extracellular FKN is cellular membrane-impermeable. We examined the effects of FKN on migratory activity and proliferation, but no effects were observed. Thus, FKN modulates the microglial activity that is induced by LPS, but the effect is limited. FKN regulates microglial migration, but it may not be effective in pro-inflammatory microglia; the FKN concentration may also be insufficient because the effect of FKN is sometimes dose-dependent [41]. In addition, Lauro et al. has shown similar results in NO production that provides FKN 24 hours after LPS treatment [42]. They show a more profound effect compared to ours, perhaps suggesting the importance of time-dependent changes in the parameters we examined.

We subsequently found that CX3CR1 mRNA was downregulated by LPS. As shown in Fig 3B, the expression of exogenous CX3CR1 possibly raises the effect of FKN. As shown in Fig 2, the decrease in CX3CR1 mRNA by LPS lasts for 24 h. The CX3CR1 ligand, FKN also suppresses CX3CR1 at the mRNA level, but in this case, it restores by 24 h [43]. Thus, some different mechanisms underlying the suppression of CX3CR1 gene expression may exist. The overexpression of CX3CR1 has been shown to result in a reduction in the concentrations of pro-inflammatory mediators in mouse brains [44, 45], showing that enhancing FKN signaling by compensating for CX3CR1 may be useful. The overexpression of CX3CR1 suppressed LPS-induced NO production without the administration of FKN. We assumed that endogenous FKN would be released from microglial BV-2 cells; however, endogenous FKN was not detectable even following LPS administration (Fig 3). We also had to consider the potential contributions by indeterminate ligand(s) to stimulate CX3CR1 in the serum of the culture media, but this was not likely because the LPS-induced NO production was not enhanced in the absence of serum (S3 Fig). Of note, we used a soluble form of FKN that works as an extracellular chemoattractant that promotes cellular functions. On the other hand, the membrane-bound form has been proposed to act as an adhesion molecule for trapping CX3CR1-expressing cells. The reduction in CX3CR1 may also decrease the adhesive properties of microglial cells.

The disruption of FKN signaling in microglia results in various neurological symptoms elicited by various forms of stress. To extend the suppression of microglial activity by the FKN signal to other inflammatory responses, pro-inflammatory stimuli were administered, and the CX3CR1 gene expression was assessed. We observed that CX3CR1 gene expression was downregulated by ATP and ethanol. Given that mice with defective CX3CR1 in microglia show various neurological symptoms under various forms of stress [11, 46], the stress-induced behavioral changes may have been partially due to the stimuli-induced CX3CR1 downregulation. In order to gain a better understanding, the mechanism underlying the downregulation of CX3CR1 gene expression by pro-inflammatory signals and its similar occurrence in human microglia need to be investigated.

In conclusion, we have demonstrated that delayed FKN administration reduces LPS-induced NO production without changing the iNOS protein concentration. In contrast, neither the migratory nor the proliferative activities were altered by FKN; thus, the effect of delayed FKN administration may be limited. We have also found that LPS and other pro-inflammatory stimuli decrease the expression of CX3CR1, the mechanism of which is still unknown, and CX3CR1 overexpression restores LPS-induced NO production. These findings suggest that the attenuation of FKN signaling is common under inflammatory conditions. Thus, compensating for the signal via the administration of FKN or the upregulation of CX3CR1 (although a method for this remains to be uncovered) may improve anti-inflammatory responses and lead this to be considered as a therapeutic targeting method.

## Supporting information

S1 Fig Identification of primary microglial cells. Primary microglial cells were isolated by shaking from mixed glial cells, and plated (see Materials and Methods). Those cells were fixed and immunostained with GFAP (green) and CD11b (red). Most of the cells were found to be CD11b-positive (microglial) cells. For DNA staining, cells were incubated with DAPI (blue).
(PDF)

S2 Fig Protein levels of iNOS, actin, and GAPDH in BV-2-treated cells. (PDF)

S3 Fig NO measurement. BV-2 cells were incubated in DMEM with either 5% FBS or 0.5% bovine serum albumin and treated with 500 ng/ml LPS for 24 h. The media were obtained, and NO production was measured by assessing nitrite levels. NO production was not enhanced without the addition of serum.
(PDF)

S1 Table Oligonucleotide primers used for the quantitative real-time PCR analysis. (PDF)
