# Supplementary figures and images for: Modulation of inflammatory responses by fractalkine signaling in microglia
Source: PLoS One. 2021 May 21;16(5):e0252118. doi: 10.1371/journal.pone.0252118 (PMC8139449; doi:10.1371/journal.pone.0252118)

**S1 Fig**

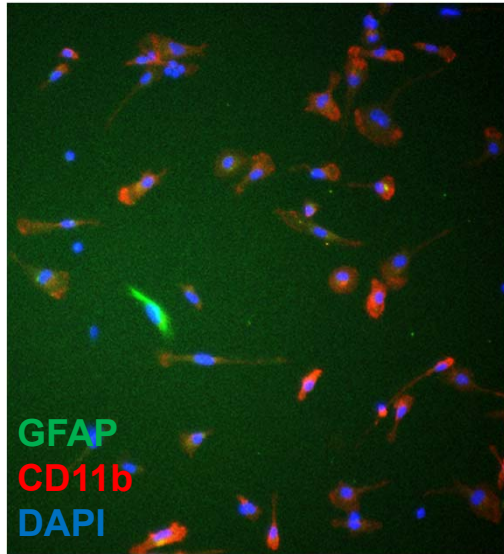

Supplement: S1 Fig — Primary microglial cells were isolated by shaking from mixed glial cells, and plated (see Materials and Methods). Those cells were fixed and immunostained with GFAP (green) and CD11b (red). Most of the cells were found to be CD11b-positive (microglial) cells. For DNA staining, cells were incubated with DAPI (blue). (PDF) [file pone.0252118.s001.pdf]

## S2 Fig

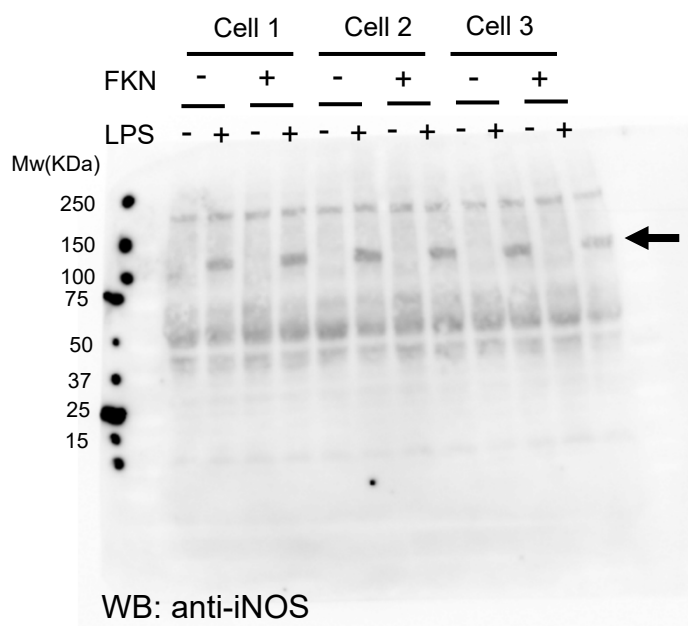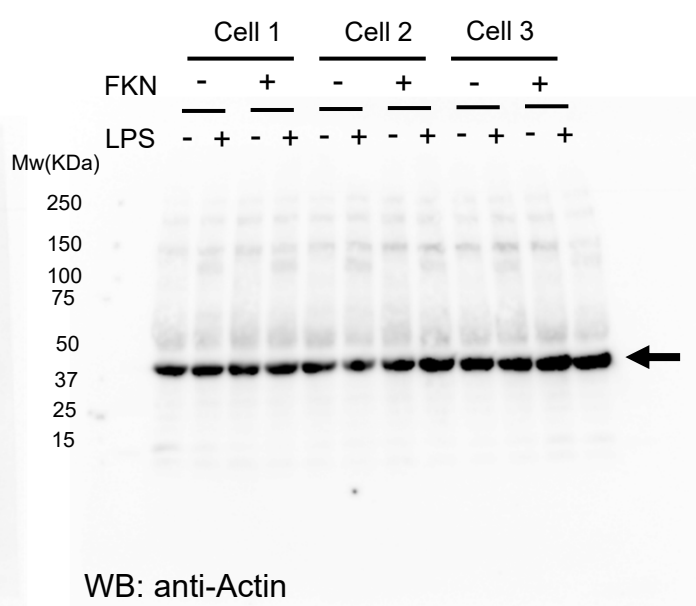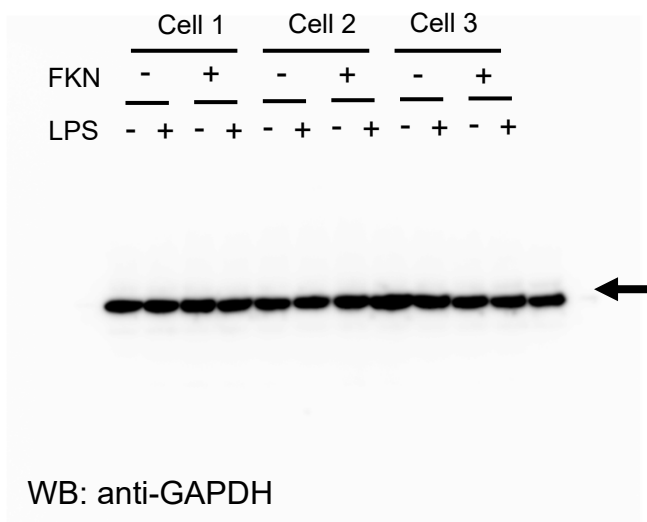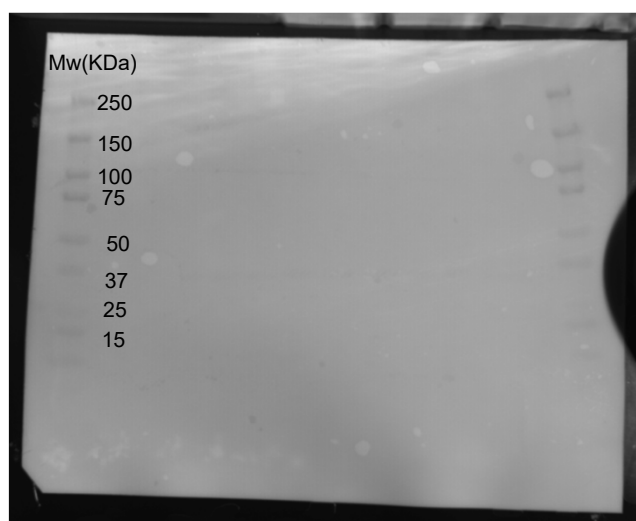

Supplement: S2 Fig — (PDF) [file pone.0252118.s002.pdf]

S3 Fig

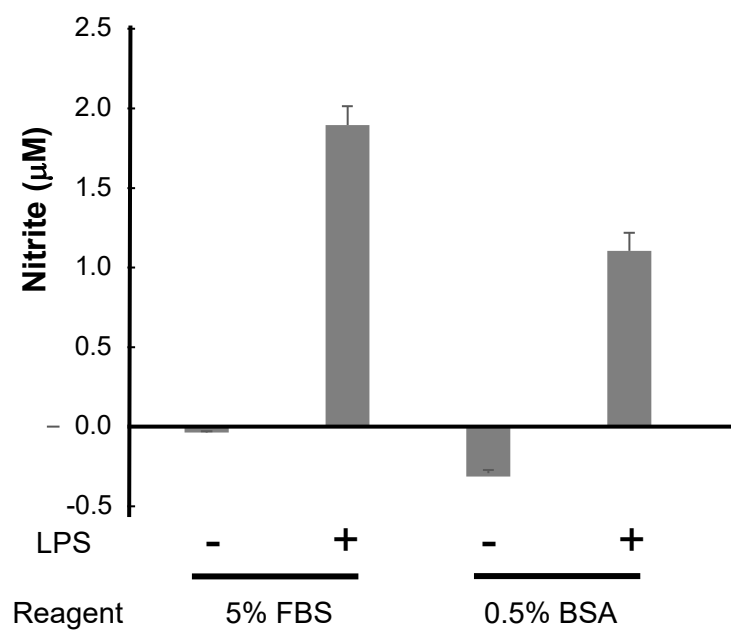

Supplement: S3 Fig — BV-2 cells were incubated in DMEM with either 5% FBS or 0.5% bovine serum albumin and treated with 500 ng/ml LPS for 24 h. The media were obtained, and NO production was measured by assessing nitrite levels. NO production was not enhanced without the addition of serum. (PDF) [file pone.0252118.s003.pdf]
