# Supplementary material for: Modulation of inflammatory responses by fractalkine signaling in microglia
Source: PLoS One. 2021 May 21;16(5):e0252118. doi: 10.1371/journal.pone.0252118 (PMC8139449; doi:10.1371/journal.pone.0252118)
Supplement: S1 Table — (PDF) [file pone.0252118.s004.pdf]

S1 Table

Gene	Forward/Reverse	Sequence	Size (bp)
GAPDH	Forward	TGCATCCTGCACCACCAACTGC	203
	Reverse	ACAGCCTTGGCAGCACCAGTGG	
β-Actin	Forward	AGCCATGTACGTAGCCATCC	228
	Reverse	CTCTCAGCTGTGGTGGTGAA	
iNOS	Forward	GGCAAACCCAAGGTCTACGTT	154
	Reverse	TCGCTCAAGTCCAGCTTGGT	
CX3CR1	Forward	CAGCATCGACCGGTACCTT	65
	Reverse	GCTGCACTGTCCGGTTGTT	
